# Impaired T Cell Responsiveness to Interleukin-6 in Hematological Patients with Invasive Aspergillosis

**DOI:** 10.1371/journal.pone.0123171

**Published:** 2015-04-02

**Authors:** Jose F. Camargo, Alyajahan Bhimji, Deepali Kumar, Rupert Kaul, Rhea Pavan, Andre Schuh, Matthew Seftel, Jeffrey H. Lipton, Vikas Gupta, Atul Humar, Shahid Husain

**Affiliations:** 1 Transplant Infectious Diseases, Multi-Organ Transplant Program, University Health Network, University of Toronto, Toronto, Ontario, Canada; 2 Department of Medicine, University Health Network, Toronto, Ontario, Canada; 3 Department of Laboratory Medicine & Pathobiology, University of Toronto, Toronto, Ontario, Canada; 4 Department of Immunology, University of Toronto, Toronto, Ontario, Canada; 5 Princess Margaret Cancer Centre, University Health Network, Toronto, Ontario, Canada; 6 Division of Medical Oncology and Hematology, University Health Network, Toronto, Ontario, Canada; Leibniz Institute for Natural Products Research and Infection Biology- Hans Knoell Institute, GERMANY

## Abstract

Invasive mold infections (IMI) are among the most devastating complications following chemotherapy and hematopoietic stem cell transplantation (HSCT), with high mortality rates. Yet, the molecular basis for human susceptibility to invasive aspergillosis (IA) and mucormycosis remain poorly understood. Herein, we aimed to characterize the immune profile of individuals with hematological malignancies (n = 18) who developed IMI during the course of chemotherapy or HSCT, and compared it to that of hematological patients who had no evidence of invasive fungal infection (n = 16). First, we measured the expression of the pattern recognition receptors pentraxin 3, dectin-1, and Toll-like receptors (TLR) 2 and 4 in peripheral blood of chemotherapy and HSCT recipients with IMI. Compared to hematological controls, individuals with IA and mucormycosis had defective expression of dectin-1; in addition, patients with mucormycosis had decreased TLR2 and increased TLR4 expression. Since fungal recognition via dectin-1 favors T helper 17 responses and the latter are highly dependent on activation of the signal transducer and activator of transcription (STAT) 3, we next used phospho-flow cytometry to measure the phosphorylation of the transcription factors STAT1 and STAT3 in response to interferon-gamma (IFN-γ) and interleukin (IL)-6, respectively. While IFN-γ/STAT1 signaling was similar between groups, naïve T cells from patients with IA, but not those with mucormycosis, exhibited reduced responsiveness to IL-6 as measured by STAT3 phosphorylation. Furthermore, IL-6 increased *Aspergillus*-induced IL-17 production in culture supernatants from healthy and hematological controls but not in patients with IA. Altogether, these observations suggest an important role for dectin-1 and the IL-6/STAT3 pathway in protective immunity against *Aspergillus*.

## Introduction

Patients with hematological malignancies, particularly those with acute myeloid leukemia, are highly susceptible to invasive mold infections (IMI) during the remission-induction phase of chemotherapy and in cases of refractory disease. Hematopoietic stem cell transplantation (HSCT) is increasingly used to treat hematologic malignancies, primary immunodeficiencies and other disorders [1]. Invasive aspergillosis (IA) is the most common fungal infection, and a leading cause of mortality in this patient population [2,3]. Mucormycosis is the second most common IMI in patients with hematological malignancies [2,3]. The estimated 12-week mortality rates for infections due to *Aspergillus spp* is 36% and as high as 64% for cases due to *Mucorales spp* [3]. Neutropenia, mucositis, steroid use, graft-versus-host disease and cytomegalovirus infection are known risk factors for IA [4]; in addition, iron overload and hyperglycemia are considered risk factors for mucormycosis [5]. However, the molecular basis for human susceptibility to invasive disease due to *Aspergillus spp* and *Mucorales spp* remain poorly understood. In the present study, we aimed to characterize the immune profile of individuals with hematological malignancies who developed IMI during the course of chemotherapy or following HSCT, and compared it to that of hematological patients who had no evidence of invasive fungal infection.

## Materials and Methods

### Ethics Statement

Blood sampling and peripheral blood mononuclear cell (PBMC) isolation were conducted with written informed consent of all study participants and approval of the local ethics committee (Research Ethics Board of the University Health Network).

### Study participants

This was a single-center cross-sectional study. Adult (18–70 years) hematological patients receiving chemotherapy or undergoing HSCT with diagnosis of probable/proven IMI due to *Aspergillus spp* (n = 14) or *Mucorales spp* (n = 4) based on the European Organization for Research and Treatment of Cancer/Invasive Fungal Infections Cooperative Group and the National Institute of Allergy and Infectious Diseases Mycoses Study Group (EORTC/MSG) Consensus Group criteria [6] were enrolled. Clinical features of the study patients and date of sample collection relative to diagnosis of IMI and date of HSCT are described in **S1 Table**. The non-invasive fungal infection (non-IFI) control group consisted of adult hematological patients (n = 16) receiving chemotherapy or undergoing HSCT who had no history or evidence of IFI (including probable/proven IMI by EORTC/MSG criteria) and had otherwise similar demographic and clinical characteristics to the IMI group (**S2 Table**). In addition to immunocompromised individuals, adult healthy volunteers were enrolled as a second control group (n = 7). If a patient was neutropenic at the time of enrollment, sample collection was deferred until white cell count recovery in order to have sufficient cells for experimental analysis.

### Cell isolation and stimulation

After blood sample collection in EDTA-containing tubes, serum samples were kept at -20°C until further analysis. PBMCs were separated by standard Ficoll-Paque (Sigma, St. Louis, MO) density gradient centrifugation and cryopreserved at -80°C until further analysis. Preparatory experiments confirmed that freeze-thaw procedures did not lead to an altered phenotype (data not shown). For immunoassay experiments, freshly thawed cells were suspended in RPMI 1640 medium supplemented with 2 mM L-glutamine, 100 U/mL of penicillin, 100 μg/mL streptomycin (all from Gibco, Paisley, UK), and 10% human pooled serum. Cells were cultured at a density of 2 x 10^6^ cells/mL in the absence or presence of the activators: Phorbol 12-Myristate 13-Acetate (50 ng/mL) (PMA; Sigma, St. Louis, MO) and ionomycin (1 μg/mL) (Life Technologies Inc., Grand Island, NY) (PMA-IO), *Aspergillus fumigatus* lysate (50 μg/mL) (Miltenyi Biotec, Auburn, CA), and recombinant human interleukin (IL)-6 (100 ng/mL) (R&D Systems, Minneapolis, MN), for 72 h in an incubator (5% CO_2_, 95% humidity, 37°C).

### Immunostaining of cell surface antigens

Expression of surface markers was assessed using standard protocols. Briefly, freshly thawed PBMCs were washed with cold staining media: phosphate buffered saline (PBS; Gibco, Life Technologies, Grand Island, NY) containing 1% bovine serum albumin (BSA; Sigma, St. Louis, MO); incubated with Fc blocking reagent (Miltenyi Biotec, Auburn, CA) for 15min at 4°C; washed again with staining media and incubated for 15 minutes at 4°C in the presence of fluorochrome-conjugated MAbs. The following markers were analyzed: TLR2-FITC, TLR4-AF700, CD14-PE-Cy7, fixed viability stain 450 (BD Biosciences, San Diego, CA); Dectin-1-APC and CD45-PE (R&D Systems, Minneapolis, MN). Appropriate Fluorescence Minus One (FMO) controls were included in all assays. After staining, cells were washed and resuspended in staining media before analysis.

### Phospho-specific flow cytometric analysis

Freshly thawed PBMCs were resuspended in serum-free, antibiotic-free RPMI 1640 media. Cells were distributed (0.5 x 10^6^cells in 100 μL per tube) to Falcon polystyrene round-bottom tubes (12 x 75 mm; Corning Incorporated, Durham, NC) and treated with IL-6 or IFN-γ (100 ng/mL) (R&D Systems, Minneapolis, MN) for 15 min at 37°C before subjecting them to phospho-specific flow cytometric analysis as described previously [7]. Briefly, after stimulation, cells were fixed by incubating in 2% PFA (BD Cytofix Fixation Buffer; BD Biosciences) for 10 min at 37°C and pelleted. They were then permeabilized by resuspending with vigorous vortexing in 300 μL ice-cold methanol. Cells were washed in staining media. Fluorophore-specific MAbs were added and incubated for 30 min at RT. The following markers were analyzed: CD3-PE/Cy7, CD4-APC/Cy7, CD45RO-PerCP/Cy5.5, CD33-APC, STAT1 (pY701)-AF488, STAT3 (pY705)-AF488, (BD Biosciences, San Diego, CA); and CD45-PE (R&D Systems, Minneapolis, MN). The cells were washed with staining media and pelleted. Finally, the samples were resuspended in 250 μL of staining media and analyzed.

### Flow cytometry acquisition and analysis

At least 10,000 gated events were collected for each sample. Singlet events were acquired based on forward scatter and side scatter properties (**S5 and S6 Figs**). Potential blast cells were excluded at the time of analysis by gating on CD45 high cells (**S5 and S6 Figs**). Dead cells were excluded on the basis of forward scatter and side scatter properties and/or live/dead stain. Cells were acquired on an LSR II instrument (BD Immunocytometry Systems, San Jose, CA) and analyzed using FlowJo analytical software (Tree star, Version 9.0.2; Ashland, OR).

### Immunoassays

The levels of PTX3, IFN-γ, and IL-17A in sera or culture supernatants were determined by cytokine-specific enzyme-linked immunosorbent assays (ELISA) according to manufacturer’s instructions (PTX3 and IL-17A kits from R&D Systems, Minneapolis, MN; IFN-γ kit from Cellestis [a Qiagen company], Valencia, CA).

### Statistical Analysis

Data was analyzed using GraphPad Prism (GraphPad Software, Inc., Version 6.0; San Diego, CA) and IBM SPSS Statistics (IBM Corp, Version 20.0: Armonk, NY). Heat maps were constructed using CIMminer available at: http://discover.nci.nih.gov/cimminer (Genomics and Bioinformatics Group, Center for Cancer Research (CCR) National Cancer Institute (NCI); Bethesda, MD). Linear regressions were completed using GraphPad via least-squares fit. When analyzing phospho-specific flow data, fold change was calculated by dividing the median fluorescence intensity (MFI) of the stimulated sample by that of the unstimulated sample. For immunoassay data, fold change was calculated by dividing the cytokine levels produced in response to stimulation by those of non-stimulated cells; or, when indicated, by dividing the cytokine levels produced in response to *Aspergillus* in the presence of IL-6 by those of cells simulated with *Aspergillus* lysate alone. Detectable *Aspergillus*-induced cytokine production was defined as >2.5 fold change from baseline. Values ≥ 3 SD were excluded from the data analysis. Statistical significance was determined using unpaired and paired Student’s *t*-tests, as well as Pearson’s Chi-Square Test. The threshold of statistical significance was defined as *p* < 0.05.

## Results and Discussion

**Fig 1:**
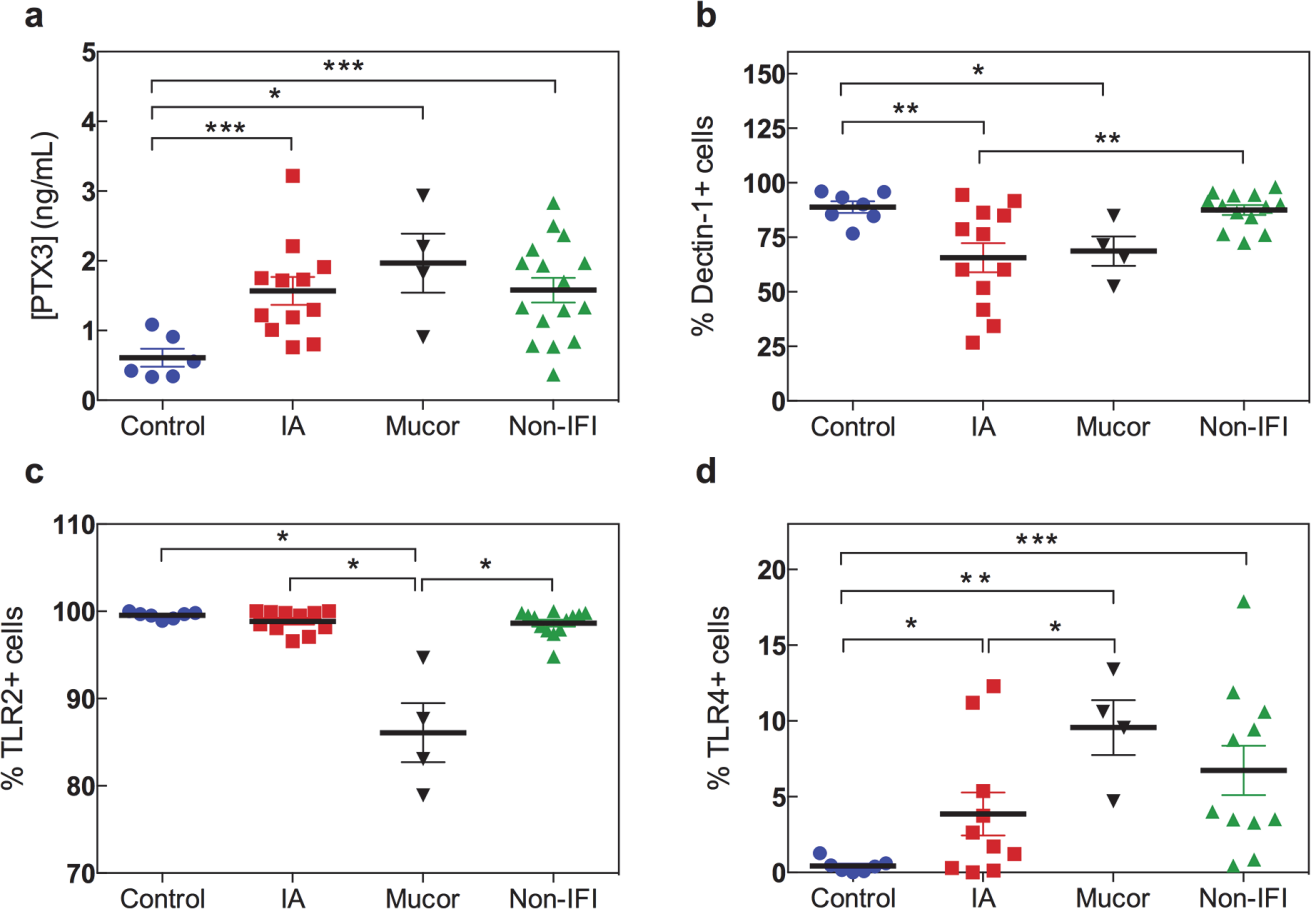
Pattern recognition receptors in hematological patients with IMI. (a) Serum levels of PTX3 (ng/mL) measured by immunoassay in healthy controls (blue circles; n = 6), hematological patients with IA (red squares; n = 12) or mucormycosis (black inverted triangles; n = 4) and non-IFI hematological controls (green triangles; n = 16) are shown. *p<0.05, **p<0.01, and ***p<0.005 using the unpaired two-tailed Student’s *t*-test. (b-d) Surface expression of dectin-1 (b), TLR2 (c) and TLR4 (d) on monocytes was measured by flow cytometry. Gating on CD45^high^CD14+ cells was performed in order to avoid interference of the analysis by potential blasts in leukemic patients with residual disease. Dot plots represent the percentage of monocytes (CD45^high^CD14+ cells) expressing dectin-1, TLR2 or TLR4 in peripheral blood samples from healthy controls (blue circles; n = 7), hematological patients with IA (red squares; n = 12) or mucormycosis (black triangles; n = 4) and non-IFI hematological controls (green triangles; n = 13). *p<0.05, **p<0.01 using the unpaired two-tailed Student’s *t*-test. All data are shown as mean ± s.e.m.

The first cellular line of defense against filamentous fungi consists of epithelial cells and phagocytes, particularly pulmonary alveolar macrophages [5,8]. Detection of fungal antigens such as beta-glucans, galactomannan and zymosan by epithelial and phagocytic cells occurs via pattern recognition receptors (PRRs), which recognize highly conserved structures expressed on the cell wall of invading fungi. Pentraxin 3 (PTX3) is a soluble PRR that is rapidly released by neutrophils and other cell types in response to inflammatory signals [9]. PTX3 has potent anti-*Aspergillus* activity [9] but its role in response to other filamentous fungi is unknown. Genetic deficiency of PTX3 is associated with increased risk of IA in HSCT recipients [10]. Circulating levels of PTX3 are elevated in hematological patients with IMI and normalize with successful antifungal therapy [11]. In our cohort, PTX3 levels were significantly higher in patients with IMI when compared to healthy controls (1.67 ± 0.72 vs. 0.61 ± 0.32, ng/mL, respectively; *p* = 0.0001) but they were similar between patients with IMI and non-IFI hematological controls (**S1A Fig**). Compared to healthy volunteers, serum PTX3 levels were significantly elevated in patients with IA (0.61 ± 0.32 vs. 1.57 ± 0.69, ng/mL, respectively; *p* = 0.0009) (**Fig 1A**). However, PTX3 levels did not discriminate between cases of IA and patients with mucormycosis (1.57 ± 0.69 vs. 1.97 ± 0.84, ng/mL, respectively; *p* = 0.43) or hematological controls (1.57 ± 0.69 vs. 1.63 ± 0. 7, ng/mL, respectively; *p* = 0.82). This observation is not surprising as PTX3 is not highly specific for *Aspergillus* infection [8].

In addition to soluble PRRs, fungal recognition by the innate immune system relies on membrane-bound PRRs. Dectin-1 and the Toll-like receptors (TLR) 2 and 4 are involved in the recognition of *Aspergillus spp* and *Mucorales spp* in humans [5,8,12]. Polymorphisms in the genes encoding dectin-1 (*CLEC7A*) and TLR4 are associated with increased susceptibility to IA in hematological patients [13–15]. To gain insight into the expression of membrane-bound PRRs in the setting of IMI, we next measured the surface levels of dectin-1, TLR2 and TLR4 on circulating monocytes using flow cytometry. Monocyte counts were similar for all the study groups (**S3 Table**). Potential blast cells were excluded at the time of analysis by gating on CD45^high^ cells (**S5 Fig**). The percentage of dectin-1-expressing CD14+ cells was significantly reduced in patients with IMI when compared to that of non-IFI hematological patients or healthy controls (66.4 ± 20.8 vs. 87.5 ± 8.12 [*p* = 0.001] or 88.9 ± 7.02 [*p* = 0.0009], respectively) (**S1B Fig**). This observation was true irrespective of the type of mold infection (**Fig 1B**): the frequency of dectin-1-expressing monocytes was reduced in both patients with IA and patients with mucormycosis compared to non-IFI hematological controls (65.6 ± 23.2 vs. 87.5 ± 8.12; *p* = 0.008, and 68.7 ± 13.4 vs. 87.5 ± 8.12; *p* = 0.06, respectively). TLR2 expression was decreased in patients with mucormycosis but not in those with IA (*p* = 0.03 for mucormycosis vs. all groups) (**Fig 1C**). The percentage of circulating monocytes expressing TLR2 was: 86.1 ± 6.77, 98.9 ± 1.23, 98.7 ± 1.44, and 99.5 ± 0.38 for mucormycosis, IA, non-IFI and healthy control groups, respectively. In contrast, when compared to healthy controls (0.43 ± 0.43), the frequency of TLR4-expressing monocytes was significantly increased in patients with mucormycosis (9.57 ± 3.63; *p* = 0.01) and, to a less extent, in those with, IA (3.86 ± 4.49; *p* = 0.04) and non-IFI hematological controls (6.75 ± 5.4; *p* = 0.003) (**Fig 1D**). Of note, all the patients with IMI due to *Mucorales spp* received therapy with liposomal amphotericin B (**S1 Table**); thus, this observation is consistent with the notion that exposure to liposomal amphotericin B upregulates TLR4 expression [16]. These findings suggest that while dectin-1 expression is profoundly reduced in both patients with IMI due to *Aspergillus spp* or *Mucorales spp*, TLR diversion from TLR2 to TLR4 is characteristic of patients with mucormycosis. Reduced dectin-1 expression in patients with IMI is consistent with studies showing that loss-of-function polymorphisms in *CLEC7A* result in increased susceptibility to IA following chemotherapy or HSCT [13,15].

Fungal recognition via PRRs is followed by antigen processing and presentation, and ultimately, activation of the host adaptive immune response orchestrated by fungus-specific CD4+ T cells [8]. Since T-cell production capacity is significantly reduced in chemotherapy and HSCT recipients [17], we first attempted to establish whether there were differences in the frequency of circulating CD4+ T cells subsets in hematological patients by IMI status. Although the absolute lymphocyte count and memory CD4+ T cell counts were similar across groups, we observed a significantly reduced number of naïve CD4+ T cells in patients with IA, but not those with mucormycosis, compared to non-IFI controls (**S3 Table**). This finding suggests that inadequate reconstitution of the naïve CD4+ T cell compartment following chemotherapy or HSCT might be associated with an increased risk of IA.

**Fig 2:**
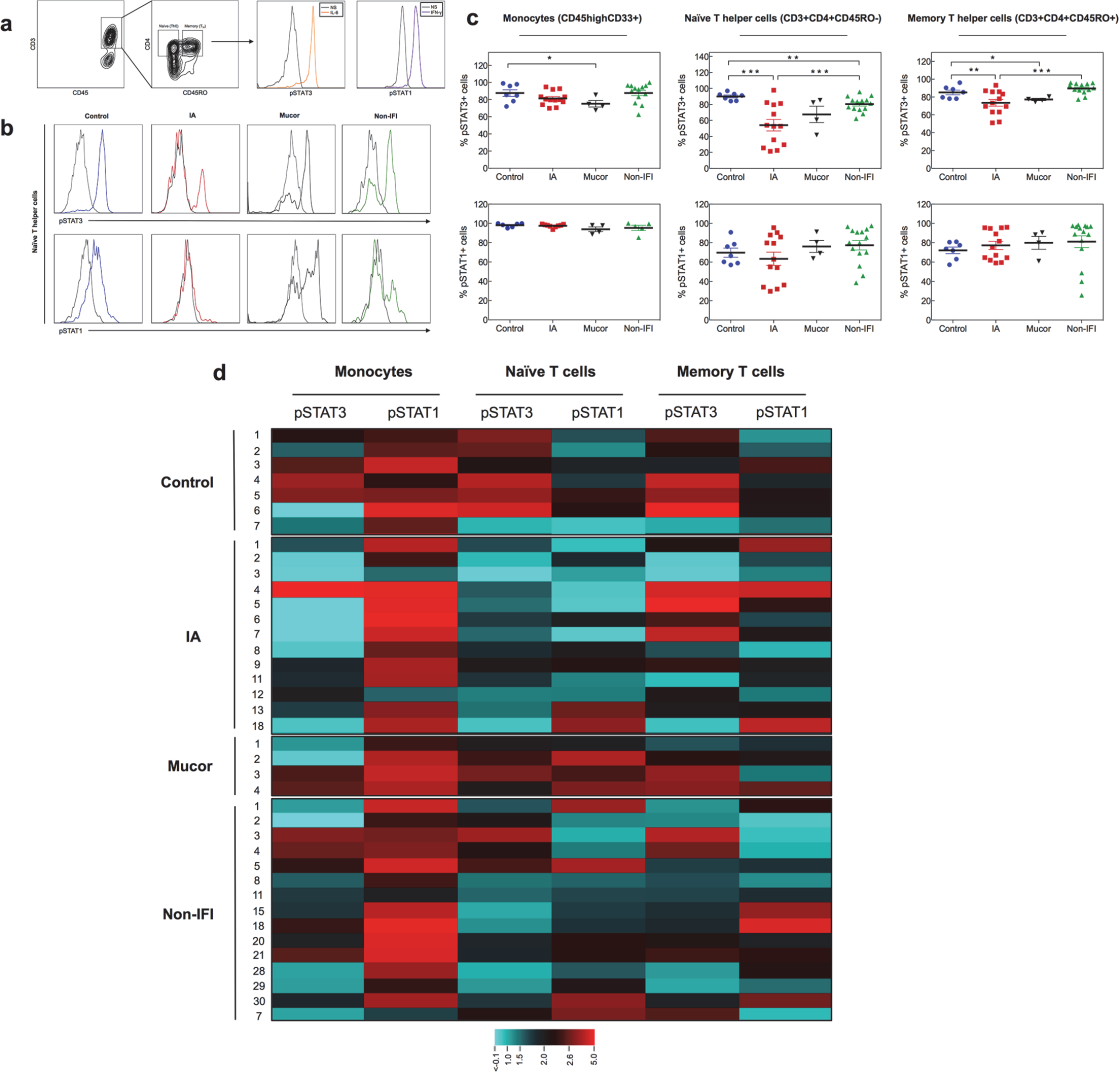
STAT1 and STAT3 phosphorylation in hematological patients with IMI. (a-d) IFN-γ-induced STAT1 phosphorylation and IL-6-induced STAT3 phosphorylation were measured in peripheral blood mononuclear cells using phospho-flow. (a) On the *left*, representative dot plots for gating of naïve and memory CD4+ T cells are shown; gating of T cells (CD3+) or monocytes (CD33+, not shown) was done on CD45^high^ cells in order to avoid interference of the analysis by potential blasts in leukemic patients with residual disease. On the *right*, representative histograms of the levels of phosphorylated STAT (pSTAT) proteins in non-stimulated (NS) and cytokine-stimulated cells are shown; baseline pSTAT levels are shown in grey, IFN-γ-induced pSTAT1 is shown in purple and IL-6-induced pSTAT3 is shown in orange. (b) Representative histograms of IL-6 induced pSTAT3 (top row) and IFN-γ-induced pSTAT1 (bottom row) in different patient groups. Y axis corresponds to number of events (i.e. number of naïve CD4+ T cells) and X axis corresponds to fluorescence intensity (i.e. pSTAT-Alexa Fluor 488). (c) Percentage of monocytes (CD45^high^CD33+ cells), naïve T helper (Th) cells (CD45^high^CD3+CD4+CD45RO- cells) and memory Th cells (CD45^high^CD3+CD4+CD45RO+ cells) expressing pSTAT3 (top row) and pSTAT1 (bottom row) in response to IL-6 and IFN-γ, respectively, in healthy controls (blue circles; n = 7), hematological patients with IA (red squares; n = 13) or mucormycosis (black inverted triangles; n = 4) and non-IFI hematological controls (green triangles; n = 15). *p<0.05, **p<0.01 and ***p<0.005 using the unpaired two-tailed Student’s *t*-test. Data are shown as mean ± s.e.m. (d) Heat map for log2 scale of mean fluorescence intensity (MFI) fold change. Fold change was calculated by dividing the MFI of the cytokine-stimulated sample by that of the unstimulated sample. Heat map color scale is showed in the bottom. Each row on the heat map corresponds to an individual patient or control as indicated by the study ID number on the left. Each column on the heat map corresponds to specific cell type/pSTAT as indicated on the top. Note reduced IL-6-induced pSTAT3 in monocytes (*p* = 0.01 for IA vs. healthy controls and *p*<0.05 for IA vs. non-IFI) and naïve CD4+ T cells (*p* = 0.006 for IA vs. healthy controls and *p* = 0.04 for IA vs. non-IFI) from patients with IA, using unpaired two-tailed Student’s *t*-test.

We next aimed to assess for qualitative defects in CD4+ T cells amongst hematological patients with IMI. T helper (Th) 17 cells play an important role in anti-fungal immunity against *Aspergillus* [8]. Since dectin-1–mediated fungal recognition favors Th17 differentiation [18,19], we hypothesized that individuals with IMI have defects in the signaling pathways that lead to Th17 differentiation. The Janus kinase/signal transducer and activator of transcription (JAK/STAT) signaling pathways have an important role in the control of innate and adaptive immune responses [20]. Assessment of JAK/STAT phosphorylation by phospho-specific flow cytometric analysis (phospho-flow) [21,22] has been shown to be a useful strategy to identify immune correlates of clinical outcomes in infectious diseases, autoimmunity and cancer [7,23–25]. The interleukin-6 (IL-6)/STAT3 and interferon-gamma (IFN-γ)/STAT1 pathways are critical for the development of Th17 cells [26] and macrophage activation, respectively. Using phospho-flow, we measured IL-6-induced STAT3 and IFN-γ-induced STAT1 phosphorylation in circulating monocytes, naïve and memory CD4+ T cells (**Fig 2A**). The percentage of naïve CD4+ T cells responding to IL-6, as measured by STAT3 phosphorylation, was significantly reduced in patients with IMI (**S2 Fig**). This observation was particularly true for patients with IA when compared to healthy and non-IFI hematological controls (54 ± 26 vs. 90 ± 4.72 [*p* = 0.0003] and 80.4 ± 9.28 [*p* = 0.004], respectively) (**Fig 2B and 2C**). The number of memory CD4+ T cells responding to IL-6 was also reduced, though to a less extent, in IA when compared to healthy and non-IFI hematological controls (**Fig 2C**). Patients with mucormycosis had reduced numbers of monocytes and memory CD4+ T cells responding to IL-6, as compared to healthy controls (**Fig 2C**). Monocyte and lymphocyte responsiveness to IFN-γ was similar across all study groups (**Fig 2C**). In addition to the reduced percentage of naïve CD4+ T cells responding to IL-6 (**Fig 2C**), the magnitude of IL-6-induced STAT3 phosphorylation on naïve CD4+ T cells was also significantly reduced in patients with IA (**Fig 2D**) but not in those with mucormycosis (fluorescence intensity fold change: 2.66 ± 0.74 vs. 6.28 ± 2.66 [*p* = 0.02] and 3.54 ± 1.31 [*p* = 0.04] for IA vs. healthy and non-IFI hematological controls, respectively). Similarly, IA cases exhibited defective monocyte responsiveness to IL-6, as measured by STAT3 phosphorylation (fluorescence intensity fold change: 2.40 ± 1.08), when compared to healthy and non-IFI controls (4.67 ±1.81 [*p* = 0.03] and 3.50 ± 1.23 [*p* = 0.04], respectively) (**Fig 2D**). These findings are consistent with the increased risk of IA previously reported in patients with autosomal dominant STAT3 deficiency [27,28].

**Fig 3:**
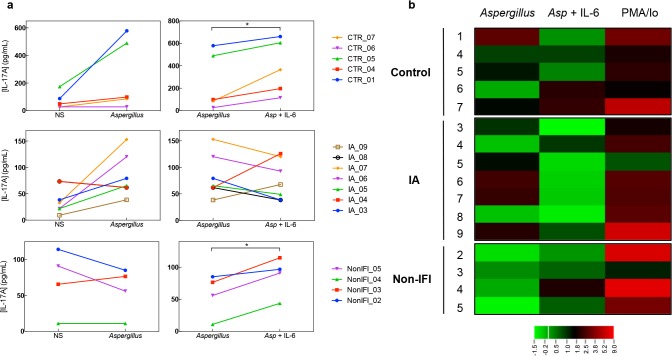
Effect of IL-6 on *Aspergillus*-induced IL-17 production. (**a**) Levels IL-17 (pg/mL) measured by immunoassay on culture supernatants are shown. *Left* panels show the levels of IL-17 in supernatant of peripheral blood mononuclear cells (PBMCs) incubated in media alone (NS) or in the presence of *Aspergillus fumigatus* lysate (50 mg/mL) for 72hr. *Right* panels correspond to levels of IL-17 in supernatant of PBMCs incubated with *Aspergillus fumigatus* alone or in the presence of recombinant human IL-6 (100ng/mL). Detectable *Aspergillus*-induced cytokine production was defined as >2.5 fold change from baseline. Each line corresponds to an individual patient or control as indicated by the study ID number on the right. *p<0.05 using the paired two-tailed Student’s *t*-test. (**b**) Heat map for log2 scale of IL-17 levels fold change. Each row on the heat map corresponds to an individual patient or control as indicated by the study ID number on the left. Fold change was calculated by dividing the IL-17 levels produced in response to Phorbol 12-Myristate 13-Acetate and ionomycin (50 ng/mL and 1 mg/mL, respectively; depicted as PMA/Io) or *Aspergillus fumigatus* (50 mg/mL; depicted as *Aspergillus*) stimulation by those of non-stimulated cells; and by dividing IL-17 levels in response to *Aspergillus* (50 mg/mL) plus IL-6 (100ng/mL) by those of cells stimulated with *Aspergillus* lysate alone (depicted as *Asp* + IL-6). Heat map color scale is showed in the bottom.

As STAT3 mutations result in impaired Th17 cell responses [29], we next evaluated cytokine production by *Aspergillus*-specific T cells. The magnitude of *Aspergillus*-induced cytokine production was variable across groups (**S3 Fig**). IFN-γ in response to *Aspergillus fumigatus* lysate was detectable in culture supernatants from 5 of 5 (100%) healthy controls, 5 of 7 (71.4%) IA cases and 1 of 4 (25%) non-IFI hematological controls (**S4 Fig**). *Aspergillus*-induced IL-17 production was detectable in 3 of 5 (60%) healthy controls, 4 of 7 (57%) IA cases, and none of the non-IFI hematological controls (**Fig 3A and 3B**). These results are consistent with the notion that among hematological patients, fungus-specific T cells are detectable only in the context of a sizeable, clinically evident, fungal burden [30]. Addition of IL-6 to the culture supernatants resulted in increased production of IL-17, as compared to *Aspergillus fumigatus* lysate alone, in healthy (388 ± 242 vs. 256 ± 257, pg/mL; *p* = 0.02) and non-IFI hematological controls (87 ± 30 vs. 57 ± 33, pg/mL; *p* = 0.016). However, this IL-17 boosting effect of IL-6 was not seen in IA patients (76 ± 37 vs. 83 ± 40, pg/mL; *p* = 0.66) (**Fig 3A**). IL-6 did not influence *Aspergillus*-induced IFN-γ production in any group (**S4 Fig**).

In summary, hematological patients with IA had an immune phenotype characterized by defective dectin-1 expression on monocytes, a depleted naïve CD4+ T cell compartment; and impaired responsiveness to IL-6 as measured by STAT3 phosphorylation on monocytes and CD4+ T cells as well as by IL-17 production in culture supernatants. A pivotal role for IL-6 in protective immunity against *Aspergillus* has been reported in mice [31] but until now, human data was lacking. Although the small number of cases prevents us from drawing any conclusions in the mucor group, patients with mucormycosis tended to have reduced dectin-1 levels and were characterized by a diversion from TRL2 to TLR4 expression. These findings improve our understanding about the immunological profile underlying susceptibility to IMI in chemotherapy and HSCT recipients. Whether these immune phenotypes have a role in prediction of clinical outcomes or constitute suitable therapeutic targets in hematological patients should be evaluated in future studies.

## Supporting Information

S1 Fig Pattern recognition receptors in hematological patients with IMI. (a) Serum levels of PTX3 (ng/mL) measured by immunoassay in healthy controls (blue circles; n = 6), hematological patients with IMI (n = 16; IA cases are shown in red squares [n = 12] and mucormycosis cases are shown in red diamonds [n = 4]) and non-IFI hematological controls (green triangles; n = 16) are shown. ***p<0.005 using the unpaired two-tailed Student’s *t*-test. (b) Expression of dectin-1, TLR2 and TLR4 was measured by flow cytometry. Representative dot plots for gating of monocytes are shown on the *left*; gating on CD45highCD14+ cells was performed in order to avoid interference of the analysis by potential blasts in leukemic patients with residual disease. Bars on the *right* represent the percentage of monocytes (CD45highCD14+ cells) expressing dectin-1, TLR2 or TLR4 in peripheral blood samples from healthy controls (blue bars; n = 7), hematological patients with IMI (red bars; n = 16) and non-IFI hematological controls (green bars; n = 13). *p<0.05 and *** *p* <0.005 using the unpaired two-tailed Student’s *t*-test. All data are shown as mean ± s.e.m.
(PDF)

S2 Fig STAT1 and STAT3 phosphorylation in hematological patients with IMI. IFN-γ-induced STAT1 phosphorylation and IL-6-induced STAT3 phosphorylation were measured in peripheral blood mononuclear cells using phospho-flow. Percentage of monocytes (CD45highCD33+ cells), naïve T helper (Th) cells (CD45highCD3+CD4+CD45RO- cells) and memory Th cells (CD45highCD3+CD4+CD45RO+ cells) expressing pSTAT3 (left panels) and pSTAT1 (right panels) in response to IL-6 and IFN-γ, respectively, in healthy controls (blue circles; n = 7), hematological patients with IMI (n = 17; IA cases shown in red squares [n = 13] and mucormycosis cases shown in red diamonds [n = 4]) and non-IFI hematological controls (green triangles; n = 14). *p<0.05, **p<0.01 and ***p<0.005 using the unpaired two-tailed Student’s *t*-test. Data are shown as mean ± s.e.m.
(PDF)

S3 Fig *Aspergillus*-induced cytokine production. IL-17 (left panels) and IFN-γ (right panels) levels were measured by immunoassay on peripheral blood mononuclear cell culture supernatants from healthy controls (n = 5), IA cases (n = 7) and non-IFI hematological controls (n = 4) after 72hr of stimulation with *Aspergillus fumigatus* lysate (50 μg/mL). Bottom panels show fold change after addition of IL-6 to the culture media calculated by dividing cytokine levels in response to *Aspergillus* (50 μg/mL) plus IL-6 (100 ng/mL) by those of cells stimulated with *Aspergillus* lysate alone. *p<0.05 using the unpaired two-tailed Student’s *t*-test. Data are shown as mean ± s.e.m.
(PDF)

S4 Fig Effect of IL-6 on *Aspergillus*-induced IFN-γ production. (a) Levels of IFN-γ (IU/mL) measured by immunoassay on culture supernatants are shown. *Left* panels show the levels of IFN-γ in peripheral blood mononuclear cells (PBMCs) incubated in media alone (NS) or in the presence of *Aspergillus fumigatus* lysate (50 μg/mL) for 72hr. *Right* panels correspond to levels of IFN-γ in PBMCs incubated with *Aspergillus fumigatus* alone or in the presence of recombinant human IL-6 (100 ng/mL). Each line corresponds to an individual patient or control as indicated by the study ID number on the right. *p<0.05 using the paired two-tailed Student’s *t*-test. (b) Heat map for log2 scale of IFN-γ levels fold change. Each row on the heat map corresponds to an individual patient or control as indicated by the study ID number on the left. Fold change was calculated by dividing the IFN-γ levels produced in response to Phorbol 12-Myristate 13-Acetate and ionomycin (50 ng/mL and 1 μg/mL, respectively; depicted as PMA/Io) or *Aspergillus fumigatus* (50 μg/mL; depicted as *Aspergillus*) stimulation by those of non-stimulated cells; and by dividing IFN-γ levels in response to *Aspergillus* (50 μg/mL) plus IL-6 (100 ng/mL) by those of cells stimulated with *Aspergillus* lysate alone (depicted as *Asp* + IL-6). Heat map color scale is showed in the bottom.
(PDF)

S5 Fig Representative gating of monocytes for surface expression of PRRs. At least 10,000 gated events were collected for each sample. Singlet events were acquired based on forward scatter and side scatter properties. Potential blast cells were excluded at the time of analysis by gating on CD45high cells. Dead cells were excluded on the basis of forward scatter and side scatter properties, and live/dead staining. The following markers were analyzed: Dectin-1-APC, TLR2-FITC, TLR4-AF700, CD14-PE-Cy7, CD45-PE, and fixed viability stain 450.
(PDF)

S6 Fig Representative gating of naïve T helper cells for phospho-flow. At least 10,000 gated events were collected for each sample. Singlet events were acquired based on forward scatter and side scatter properties. Potential blast cells were excluded at the time of analysis by gating on CD45high cells. Dead cells were excluded on the basis of forward scatter and side scatter properties. The following markers were analyzed: CD3-PE/Cy7, CD4-APC/Cy7, CD45RO-PerCP/Cy5.5, CD33-APC, CD45-PE and STAT1 (pY701)-AF488 or STAT3 (pY705)-AF488.
(PDF)

S1 Table Clinical features of patients. (PDF)

S2 Table Demographic and selected clinical features by study group. (PDF)

S3 Table White blood cell counts and mononuclear cell subpopulations for study patients. (PDF)

S4 Table Dectin-1 and pSTAT3 levels by time of sample collection. (PDF)
