# Supplementary figures and images for: Impaired T Cell Responsiveness to Interleukin-6 in Hematological Patients with Invasive Aspergillosis
Source: PLoS One. 2015 Apr 2;10(4):e0123171. doi: 10.1371/journal.pone.0123171 (PMC4383538; doi:10.1371/journal.pone.0123171)

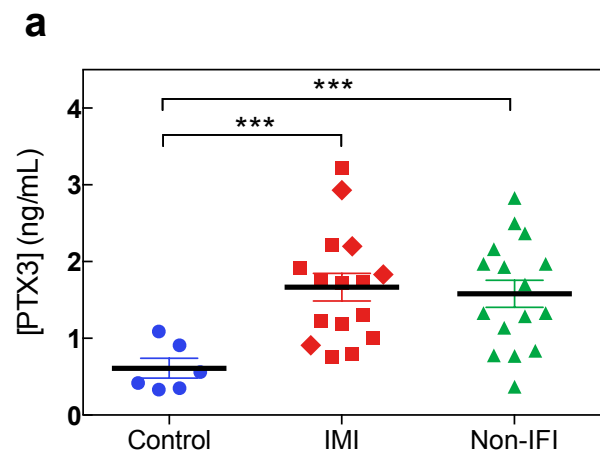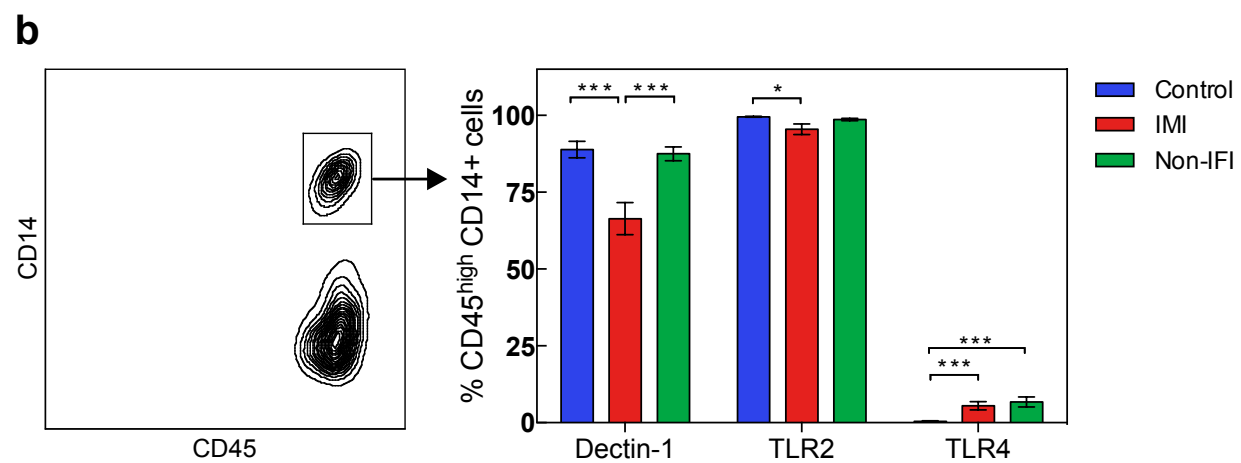

Supplement: S1 Fig — (a) Serum levels of PTX3 (ng/mL) measured by immunoassay in healthy controls (blue circles; n = 6), hematological patients with IMI (n = 16; IA cases are shown in red squares [n = 12] and mucormycosis cases are shown in red diamonds [n = 4]) and non-IFI hematological controls (green triangles; n = 16) are shown. ***p<0.005 using the unpaired two-tailed Student’s t-test. (b) Expression of dectin-1, TLR2 and TLR4 was measured by flow cytometry. Representative dot plots for gating of monocytes are shown on the left; gating on CD45highCD14+ cells was performed in order to avoid interference of the analysis by potential blasts in leukemic patients with residual disease. Bars on the right represent the percentage of monocytes (CD45highCD14+ cells) expressing dectin-1, TLR2 or TLR4 in peripheral blood samples from healthy controls (blue bars; n = 7), hematological patients with IMI (red bars; n = 16) and non-IFI hematological controls (green bars; n = 13). *p<0.05 and *** p <0.005 using the unpaired two-tailed Student’s t-test. All data are shown as mean ± s.e.m. (PDF) [file pone.0123171.s001.pdf]

### Naïve T helper cells (CD3+CD4+CD45RO-)

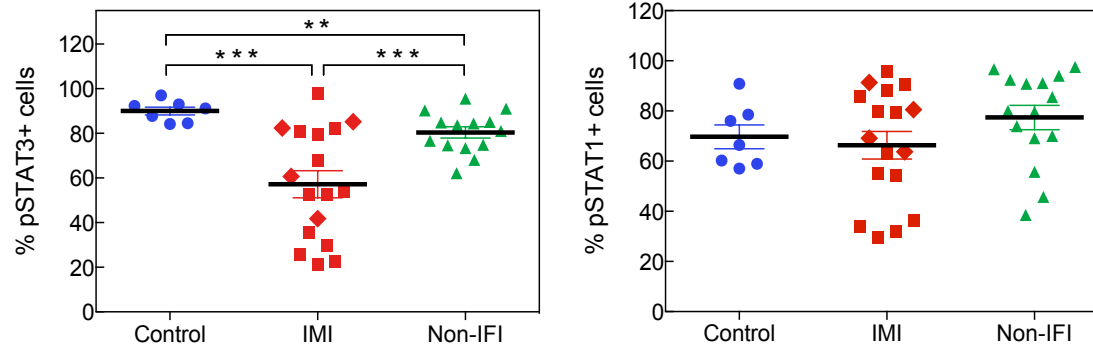

### Memory T helper cells (CD3+CD4+CD45RO+)

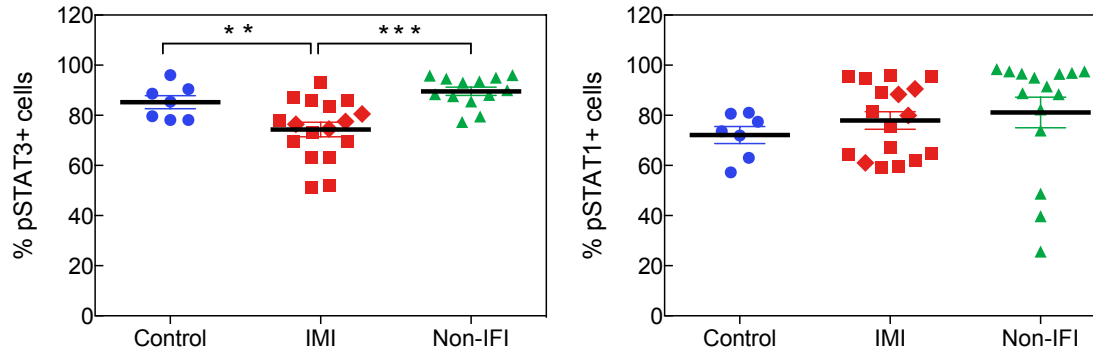

### Monocytes (CD45<sup>high</sup>CD33+)

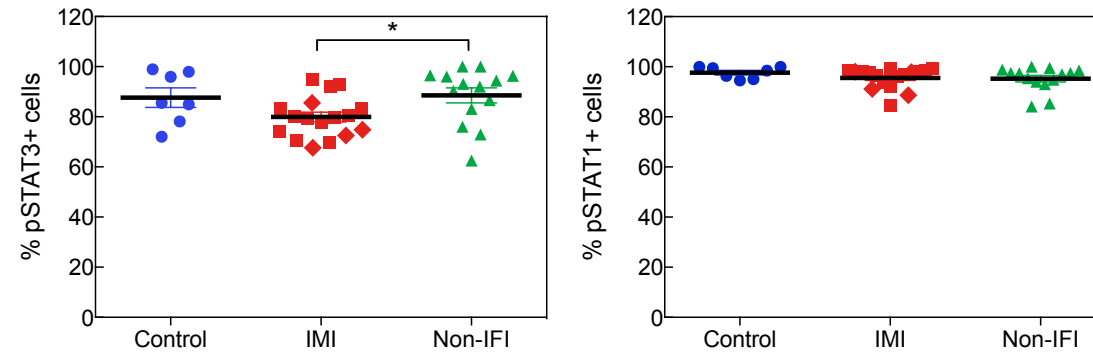

Supplement: S2 Fig — IFN-γ-induced STAT1 phosphorylation and IL-6-induced STAT3 phosphorylation were measured in peripheral blood mononuclear cells using phospho-flow. Percentage of monocytes (CD45highCD33+ cells), naïve T helper (Th) cells (CD45highCD3+CD4+CD45RO- cells) and memory Th cells (CD45highCD3+CD4+CD45RO+ cells) expressing pSTAT3 (left panels) and pSTAT1 (right panels) in response to IL-6 and IFN-γ, respectively, in healthy controls (blue circles; n = 7), hematological patients with IMI (n = 17; IA cases shown in red squares [n = 13] and mucormycosis cases shown in red diamonds [n = 4]) and non-IFI hematological controls (green triangles; n = 14). *p<0.05, **p<0.01 and ***p<0.005 using the unpaired two-tailed Student’s t-test. Data are shown as mean ± s.e.m. (PDF) [file pone.0123171.s002.pdf]

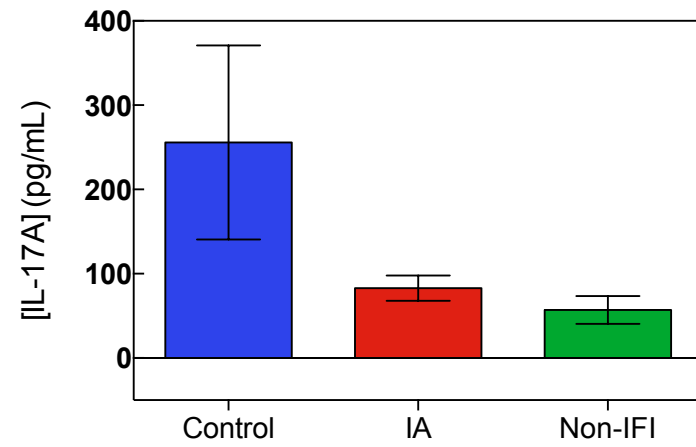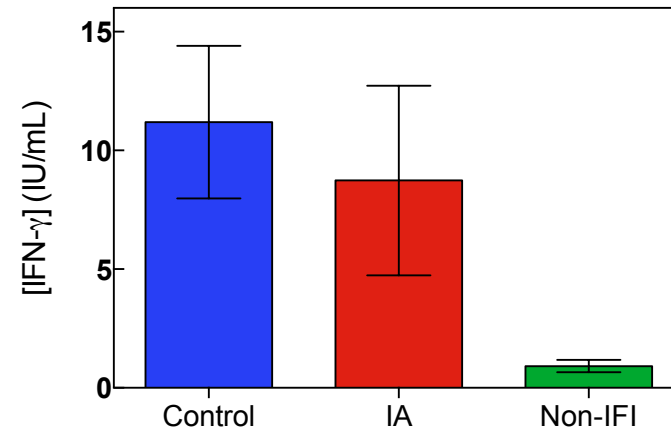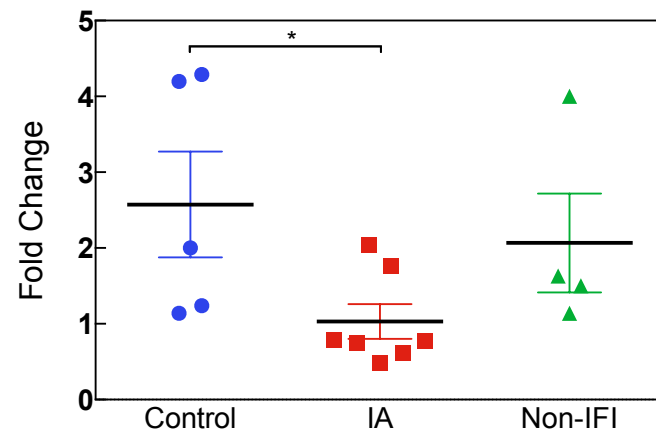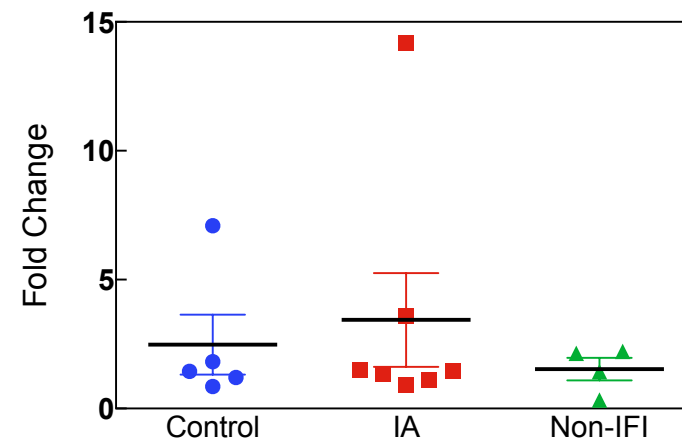

Supplement: S3 Fig — IL-17 (left panels) and IFN-γ (right panels) levels were measured by immunoassay on peripheral blood mononuclear cell culture supernatants from healthy controls (n = 5), IA cases (n = 7) and non-IFI hematological controls (n = 4) after 72hr of stimulation with Aspergillus fumigatus lysate (50 μg/mL). Bottom panels show fold change after addition of IL-6 to the culture media calculated by dividing cytokine levels in response to Aspergillus (50 μg/mL) plus IL-6 (100 ng/mL) by those of cells stimulated with Aspergillus lysate alone. *p<0.05 using the unpaired two-tailed Student’s t-test. Data are shown as mean ± s.e.m. (PDF) [file pone.0123171.s003.pdf]

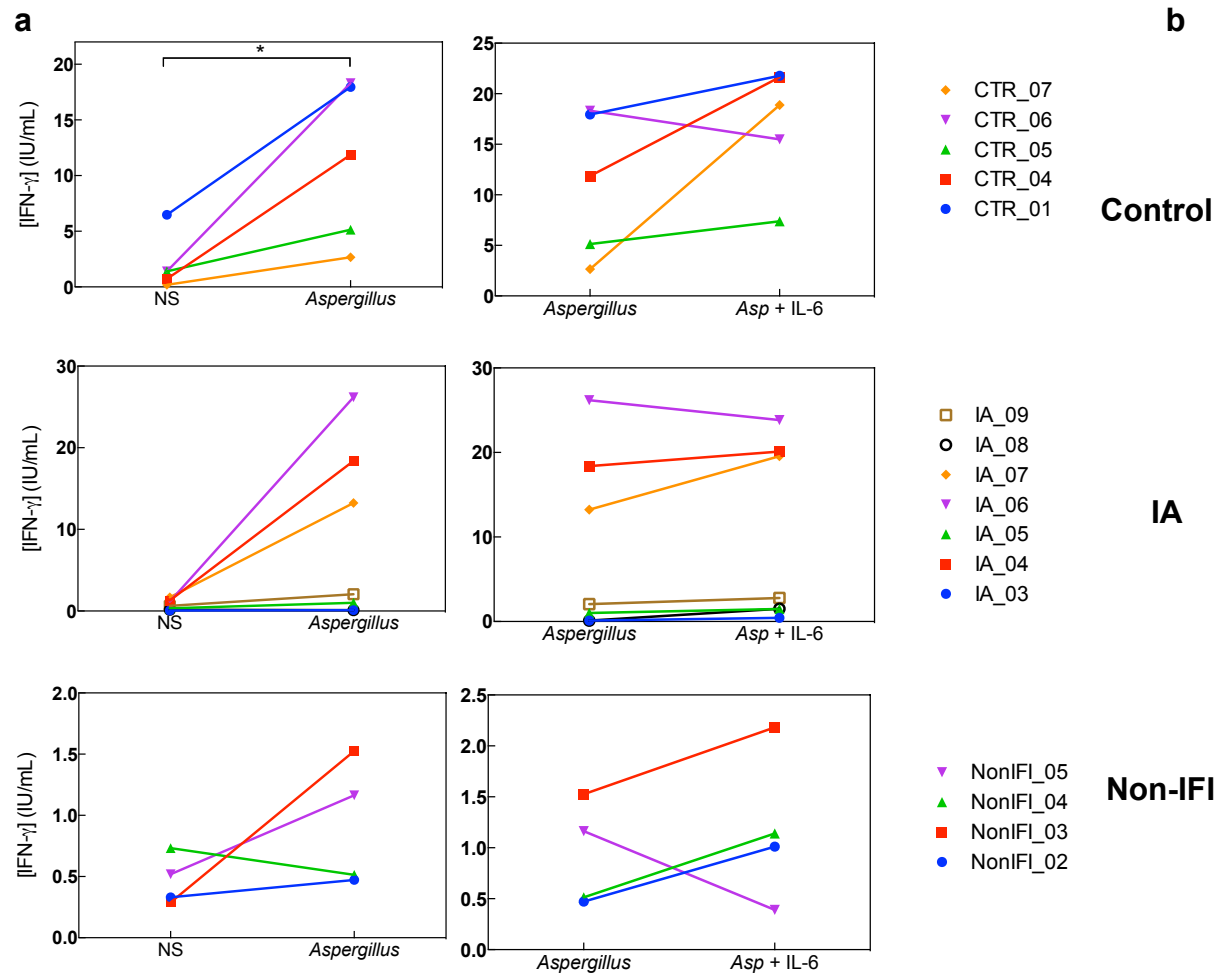

Supplement: S4 Fig — (a) Levels of IFN-γ (IU/mL) measured by immunoassay on culture supernatants are shown. Left panels show the levels of IFN-γ in peripheral blood mononuclear cells (PBMCs) incubated in media alone (NS) or in the presence of Aspergillus fumigatus lysate (50 μg/mL) for 72hr. Right panels correspond to levels of IFN-γ in PBMCs incubated with Aspergillus fumigatus alone or in the presence of recombinant human IL-6 (100 ng/mL). Each line corresponds to an individual patient or control as indicated by the study ID number on the right. *p<0.05 using the paired two-tailed Student’s t-test. (b) Heat map for log2 scale of IFN-γ levels fold change. Each row on the heat map corresponds to an individual patient or control as indicated by the study ID number on the left. Fold change was calculated by dividing the IFN-γ levels produced in response to Phorbol 12-Myristate 13-Acetate and ionomycin (50 ng/mL and 1 μg/mL, respectively; depicted as PMA/Io) or Aspergillus fumigatus (50 μg/mL; depicted as Aspergillus) stimulation by those of non-stimulated cells; and by dividing IFN-γ levels in response to Aspergillus (50 μg/mL) plus IL-6 (100 ng/mL) by those of cells stimulated with Aspergillus lysate alone (depicted as Asp + IL-6). Heat map color scale is showed in the bottom. (PDF) [file pone.0123171.s004.pdf]
