# Supplementary material for: Impaired T Cell Responsiveness to Interleukin-6 in Hematological Patients with Invasive Aspergillosis
Source: PLoS One. 2015 Apr 2;10(4):e0123171. doi: 10.1371/journal.pone.0123171 (PMC4383538; doi:10.1371/journal.pone.0123171)
Supplement: S5 Fig — At least 10,000 gated events were collected for each sample. Singlet events were acquired based on forward scatter and side scatter properties. Potential blast cells were excluded at the time of analysis by gating on CD45high cells. Dead cells were excluded on the basis of forward scatter and side scatter properties, and live/dead staining. The following markers were analyzed: Dectin-1-APC, TLR2-FITC, TLR4-AF700, CD14-PE-Cy7, CD45-PE, and fixed viability stain 450. (PDF) [file pone.0123171.s005.pdf]

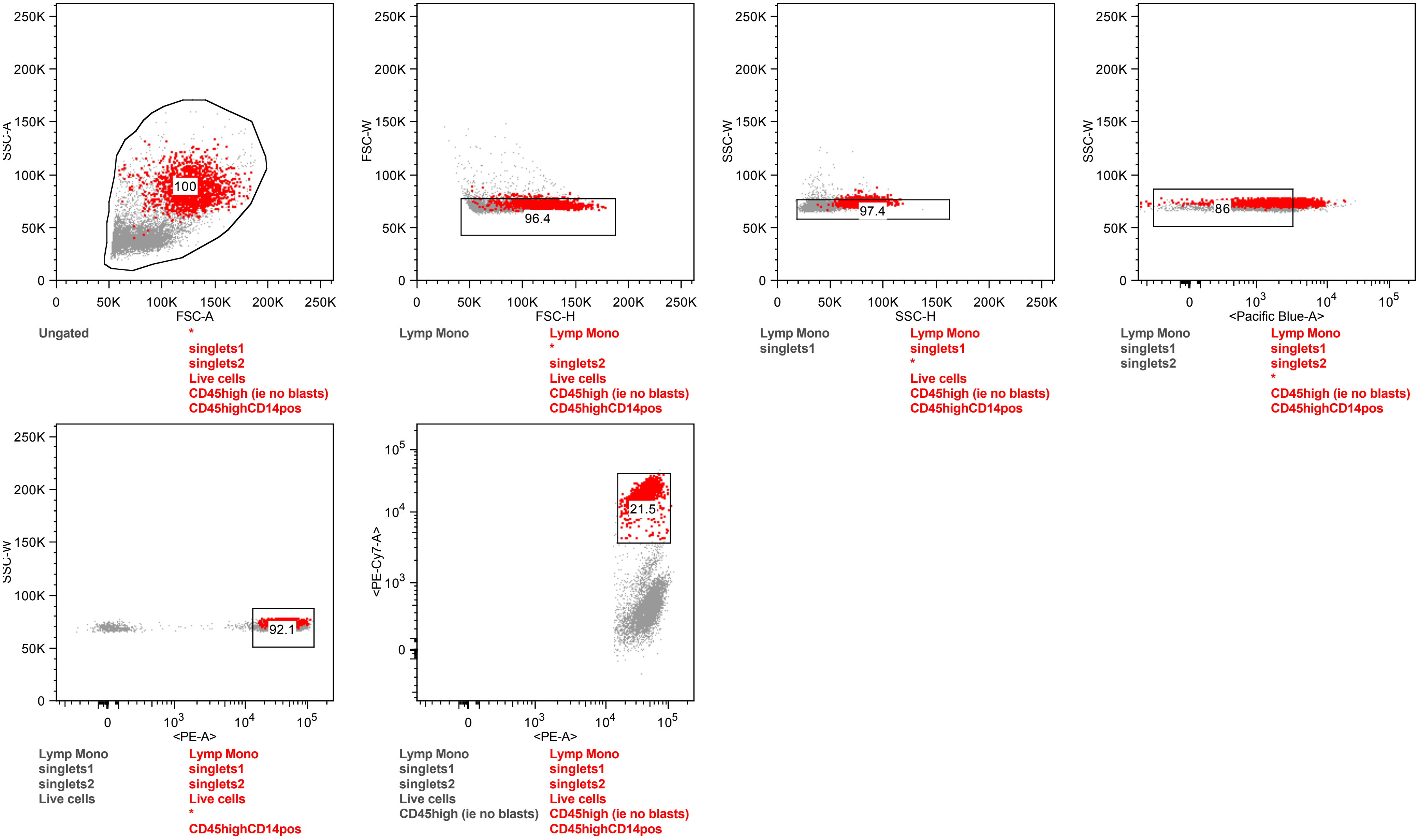

SSC-A
FSC-A
100
Ungated
*
singlets1
singlets2
Live cells
CD45high (ie no blasts)
CD45highCD14pos
FSC-W
FSC-H
96.4
Lymp Mono
Lymp Mono
*
singlets2
Live cells
CD45high (ie no blasts)
CD45highCD14pos
SSC-W
SSC-H
97.4
Lymp Mono
singlets1
Lymp Mono
singlets1
*
Live cells
CD45high (ie no blasts)
CD45highCD14pos
SSC-W
<Pacific Blue-A>
86
Lymp Mono
singlets1
singlets2
Lymp Mono
singlets1
singlets2
*
CD45high (ie no blasts)
CD45highCD14pos
SSC-W
<PE-A>
92.1
Lymp Mono
singlets1
singlets2
Live cells
Lymp Mono
singlets1
singlets2
Live cells
*
CD45highCD14pos
<PE-Cy7-A>
<PE-A>
21.5
Lymp Mono
singlets1
singlets2
Live cells
CD45high (ie no blasts)
Lymp Mono
singlets1
singlets2
Live cells
CD45high (ie no blasts)
CD45highCD14pos
