# Supplementary material for: Impaired T Cell Responsiveness to Interleukin-6 in Hematological Patients with Invasive Aspergillosis
Source: PLoS One. 2015 Apr 2;10(4):e0123171. doi: 10.1371/journal.pone.0123171 (PMC4383538; doi:10.1371/journal.pone.0123171)
Supplement: S6 Fig — At least 10,000 gated events were collected for each sample. Singlet events were acquired based on forward scatter and side scatter properties. Potential blast cells were excluded at the time of analysis by gating on CD45high cells. Dead cells were excluded on the basis of forward scatter and side scatter properties. The following markers were analyzed: CD3-PE/Cy7, CD4-APC/Cy7, CD45RO-PerCP/Cy5.5, CD33-APC, CD45-PE and STAT1 (pY701)-AF488 or STAT3 (pY705)-AF488. (PDF) [file pone.0123171.s006.pdf]

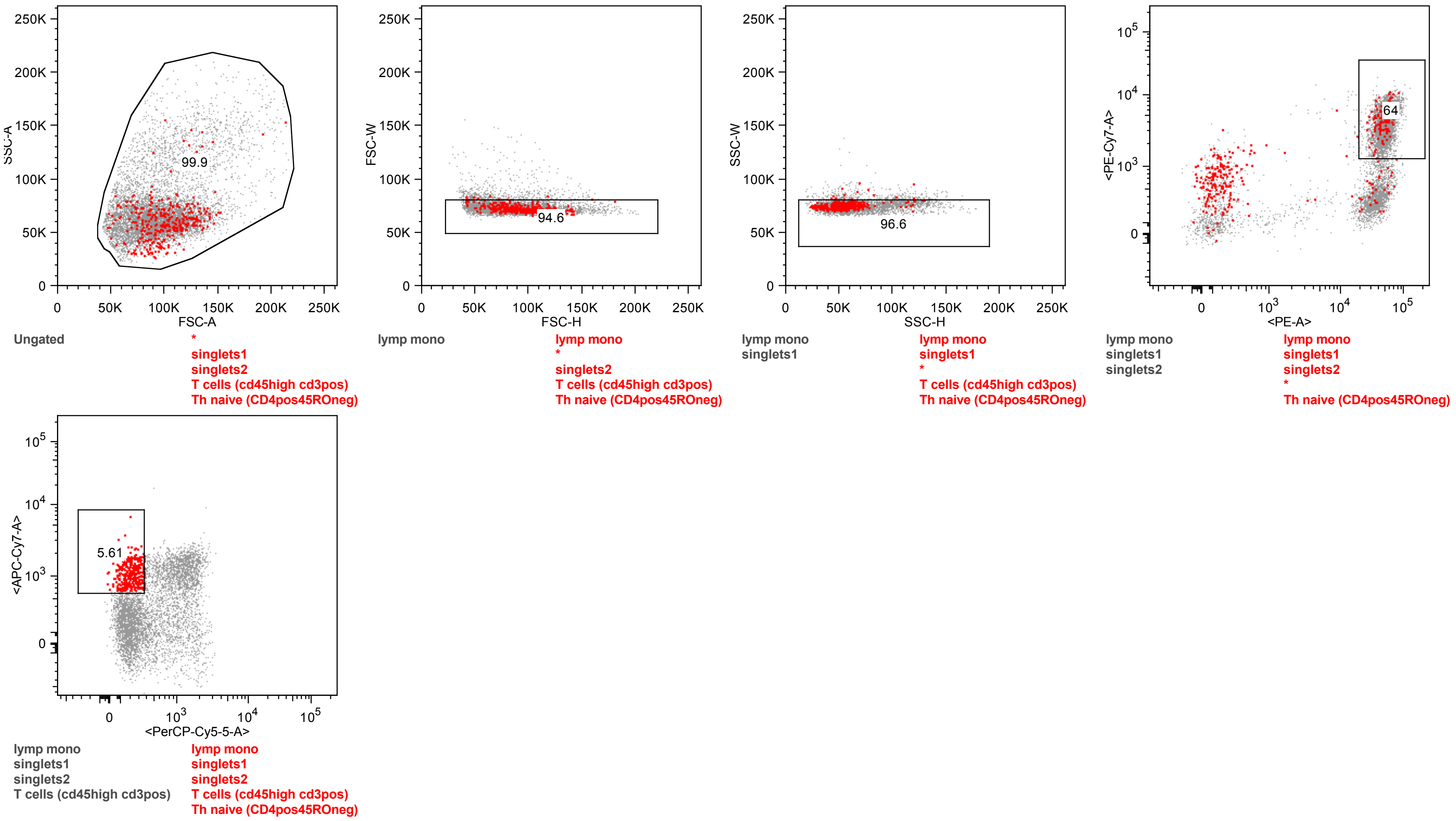

SSC-A
FSC-A
99.9
Ungated
*
singlets1
singlets2
T cells (cd45high cd3pos)
Th naive (CD4pos45ROneg)
FSC-W
FSC-H
94.6
lymp mono
lymp mono
*
singlets2
T cells (cd45high cd3pos)
Th naive (CD4pos45ROneg)
SSC-W
SSC-H
96.6
lymp mono
singlets1
lymp mono
singlets1
*
T cells (cd45high cd3pos)
Th naive (CD4pos45ROneg)
<PE-Cy7-A>
<PE-A>
64
lymp mono
singlets1
singlets2
lymp mono
singlets1
singlets2
*
Th naive (CD4pos45ROneg)
<APC-Cy7-A>
<PerCP-Cy5-5-A>
5.61
lymp mono
singlets1
singlets2
T cells (cd45high cd3pos)
lymp mono
singlets1
singlets2
T cells (cd45high cd3pos)
Th naive (CD4pos45ROneg)
