# Supplementary material for: Impaired T Cell Responsiveness to Interleukin-6 in Hematological Patients with Invasive Aspergillosis
Source: PLoS One. 2015 Apr 2;10(4):e0123171. doi: 10.1371/journal.pone.0123171 (PMC4383538; doi:10.1371/journal.pone.0123171)
Supplement: S1 Table — (PDF) [file pone.0123171.s007.pdf]

Supporting Table 1. Clinical features of study patients							
**Study ID**	**Age, years**	**Sex**	**Hematological malignancy**	**Disease status**	**Chemotherapy***	**Type of HSCT†**	**Risk factors for IMI****
**IA_001**	38	F	AML	Relapse	IT ARA-C, IT hydrocortisone	Double cord blood	GVHD, steroids, umbilical cord, CMV
**IA_002**	47	M	B-ALL	Relapse	Dana-Farber 2009>>>Hyper-CVAD	n/a	Neutropenia, steroids
**IA_003**	66	M	AML	Relapse	3+7>>>FLAG-IDA	n/a	Neutropenia
**IA_004**	64	F	MDS	Remission	ATG, CsA 2009	Matched-unrelated HSCT	GVHD, steroids
**IA_005**	49	F	AML	Remission	Azacitidine in 2012	Matched related HSCT	GVHD, steroids
**IA_006**	69	F	AML	Remission	FLAG-IDA 2013	n/a	Neutropenia
**IA_007°**	55	F	AML	Remission	NOVE-HiDAC 2010	Match-unrelated HSCT	GVHD, CMV
**IA_008**	57	M	AML	Not in remission	FLAG-IDA	n/a	Neutropenia
**IA_009**	72	M	Lymphoma>>>AML	Not in remission	Alemtuzumab/CHOP 2010 >>> FLAG-IDA	n/a	Neutropenia
**IA_010**	61	M	Relapsed AML	Remission	FLAG-IDA	Match-unrelated HSCT 2009	Neutropenia
**IA_011**	64	M	CMML>AML	Remission	NOVE-HiDAC>>> 3+7	n/a	Neutropenia
**IA_012**	34	M	CMML	Remission	Hydroxyurea	Matched-related HSCT	GVHD, steroids, CMV
**IA_013**	61	F	B-ALL	Not in remission	Vincristine, MTX	n/a	-
**IA_018**	70	M	AML	Remission	FLAG-IDA	n/a	Neutropenia
**Non-IFI_001**	44	M	AML	Remission	3+7 induction, ARA-C consolidation	n/a	Neutropenia, CMV
**Non-IFI_002**	33	F	B-ALL	Remission	Dana-Farber	n/a	Neutropenia
**Non-IFI_003**	62	M	AML (mixed phenotype)	Remission	Dana-Farber	n/a	Neutropenia
**Non-IFI_004**	43	F	AML	Remission	3+7>>>FLAG-IDA	Match-unrelated HSCT	GVHD, CMV
**Non-IFI_005**	48	M	NHL	Remission	R-CHOP>>>GDP salvage ttx	Autologus	Neutropenia

**Non-IFI_007**	23	M	B-ALL	Relapse	Dana-Farber >>>MTX	n/a	Neutropenia
**Non-IFI_008**	51	F	ALL	Remission	Dana-Farber plus DASATINIB (without L-ASPARAGINASE) 2013	Matched related HSCT	GVHD , steroids, neutropenia
**Non-IFI_011**	68	M	MDS	Remission	Azacytidine 2011	Matched-unrelated HSCT	GVHD, steroids, neutropenia
**Non-IFI_015**	46	M	AML	Remission	3+7 (2011)	Matched unrelated HSCT	Chronic GVHD, steroids, CMV
**Non-IFI_018**	42	M	B-ALL	Not in remission	Dana Farber protocol	n/a	Neutropenia
**Non-IFI_020**	53	M	B-ALL	Not in remission	Dana-Farber protocol	n/a	Neutropenia, CMV
**Non-IFI_022**	41	F	AML	Remission	3+7	n/a	Neutropenia
**Non-IFI_028**	45	F	AML	Remission	3+7	n/a	Neutropenia
**Non-IFI_029**	50	M	AML	Remission	3+7>>>FLAG_IDA	n/a	Neutropenia
**Non-IFI_030**	38	F	AML	Not in remission	3+7>>>FLAG_IDA	n/a	Neutropenia
**Non-IFI_031**	55	M	AML	Remission	3+7	n/a	Neutropenia, mucositis
**Mucor_001**	65	M	AML/MDS	Remission	Pred, MTX, imuran>>>azacytidine	n/a	Neutropenia
**Mucor_002**	25	M	ALL	Remission	DFCI	n/a	Neutropenia
**Mucor_003**	49	M	AML	Relapse	3+7>>>FLAG-IDA	n/a	Neutropenia
**Mucor_004**	58	M	AML	Remission	3+7	n/a	Neutropenia

Supporting Table 1. Clinical features of study patients (continued)									
**Study ID**	**Serum GM**	**BAL GM**	**BAL culture**	**Cytology/ Pathology**	**CT chest findings**	**Antifungal therapy**	**Antifungal at the time of sample collection**	**Time from IMI to sample collection (days)**	**Time from HSCT to sample collection (months)**
**IA_001**	n/a	Positive	*A. fumigatus*		Nodules, consolidation	VOR > CAS > L-AMB	VOR	11	13
**IA_002**	n/a	Positive	No growth		Nodules, pleural effusion	VOR	VOR	48	
**IA_003**	Positive	Positive	No growth		Nodules	VOR > CAS > L-AMB	AMB	42	
**IA_004**	Negative	Negative	*A. niger*		Nodule, halo sign+	VOR	VOR	70	56
**IA_005**	Negative	Negative	*A. fumigatus*		bilateral opacities	VOR	VOR	57	26
**IA_006**	Negative	Positive	No growth		Nodules	VOR	None	315	
**IA_007°**	Negative	Negative	*A. fumigatus* *Zygomycetes*	Septate hyphae on BAL	GGO	VOR > POS > CAS	None	389	41
**IA_008**	Negative	Positive	No growth		Nodules	VOR	VOR	11	
**IA_009**	Negative	Positive	*A. fumigatus*	Septate hyphae on BAL	Nodules	CAS > L-AMB > VOR	AMB	2	
**IA_010**	Positive	Positive	No growth		Nodules	VOR > L-AMB	AMB	16	58
**IA_011**	Negative	Positive	No growth		GGO, cavitary mass	VOR	VOR	70	
**IA_012**	Negative	Positive	*A. niger*		Nodule, cavitary lesion	L-AMB	AMB	11	16.5
**IA_013**	Negative	Positive	No growth		Consolidation	VOR	VOR	20	

					b/l, GGO				
**IA_018**	Negative	Positive	No growth		Pulmonary nodules, GGO, halo sign+	VOR	VOR	23	
**NonIFI_001**	Negative	nd	nd		Normal	None	None		
**NonIFI_002**	nd	nd	nd		Normal	MIC	MIC (prophylaxis)		
**NonIFI_003**	Negative	nd	nd		nd	None	None		
**NonIFI_004**	Negative	Negative	No growth		Small nodules	None	None		11
**NonIFI_005**	nd	nd	nd		Nd	None	None		7
**NonIFI_007**	nd	nd	nd		Small nodules	VOR > CAS	CAS (empiric)		
**Non-IFI_008**	nd	nd	nd		Small area of GGO	None	None		9
**Non-IFI_011**	nd	nd	nd		Mild heterogeneity of the lung parenchyma in keeping with GVHD	None	None		37.5
**Non-IFI_015**	nd	nd	nd		Normal	None	None		36
**Non-IFI_018**	nd	nd	nd		Small nodules	None	None		
**Non-IFI_020**	nd	nd	nd		GGO	None	None		
**Non-IFI_022**	nd	nd	nd		Normal	None	None		
**Non-IFI_028**	nd	nd	nd		Stable small solitary nodule	None	None		
**Non-IFI_029**	nd	nd	nd		Stable small solitary nodule	None	None		
**Non-IFI_030**	nd	nd	nd		Stable 3mm	None	None		

					nodules				
**Non-IFI_031**	Nd	Nd	Nd		Normal	None	None		
**Mucor_001**	Negative	nd	nd	Mucor	Nodules, GGO	L-AMB	AMB	23	
**Mucor_002**	Nd	nd	nd	Mucor	Normal	L-AMB	AMB	17	
**Mucor_003**	Negative	Negative	No growth	Mucor	opacities, nodules	L-AMB >POS	POS	177	
**Mucor_004**	Negative	nd	nd	Mucor (*Rhizopus*)	Normal	L-AMB > POS	POS	125	

IA, invasive aspergillosis; IFI, invasive fungal infection; Mucor, Mucormycosis; AML, acute myeloid leukemia; ALL, acute lymphoblastic leukemia; CMML, chronic myelomonocytic leukemia; NHL, non-Hodgkin's lymphoma; MDS, myelodysplastic syndrome; HSCT, hematopoietic stem cell transplant; ATG, anti-thymoglobulin; CsA, Cyclosporine; GM, galactomannan; GVHD, graft-versus-host disease, CMV, cytomegalovirus; GGO, ground glass opacities; VOR, voriconazole; CAS, caspofungin; L-AMB, liposomal Amphotericin B; MIC, micafungin; POS, posaconazole; n/a, not applicable; nd, not done

°Mixed IFI: this patient had recurrent episodes of IA and in addition to *Aspergillus fumigatus* also grew *Zygomycetes* in a recent bronchoalveolar lavage.
*Most recent chemotherapy regimen. 3+7= Daunorubicin, cytarabine (Ara-c); FLAG-IDA: Idarubicin, Fludarabine and Ara-c; Hyper-CVAD: Cyclophosphamide, Doxorubicin, Vincristine, Daunorubicin, Dexamethasone plus CNS prophylaxis: intrethecal (IT) Methotrexate, Ara-C; DFCI: Vincristine, Doxorubicin, MTX, methylprednisolone plus intrathecal Ara-C, MTX, hydrocortisone; NOVE-HIDAC: Mitoxantrone, Etoposide, Ara-c; R-CHOP: Rituximab, Cyclophosphamide, Doxorubicin, Vincristine, prednisolone (pred). GDP: Gemcitabine, Dexamethasone and Cisplatin.
**CMV reactivation defined as blood CMV DNA level in peripheral blood >100IU/ml in two occasions within the 12 months prior to data of sample collection.
†Full chimerism, defined as >95% of cells of donor origin, was documented for all the HSCT recipients, except IA_010 who had evidence of mixed chimerism, and IA_001 and non-IFI_011 in whom chimerism data was not available.
