# Supplementary material for: Impaired T Cell Responsiveness to Interleukin-6 in Hematological Patients with Invasive Aspergillosis
Source: PLoS One. 2015 Apr 2;10(4):e0123171. doi: 10.1371/journal.pone.0123171 (PMC4383538; doi:10.1371/journal.pone.0123171)
Supplement: S2 Table — (PDF) [file pone.0123171.s008.pdf]

Supporting Table 2. Demographic and selected clinical features by study group			
	IMI† (n=18)	Non-IFI (n=16)	χ^2, df (*P* value)
**Age**<br>Older than 40 years, n (%)	15 (83.3)	13 (81.2)	**0.025, 1 (0.874)**
**Sex**<br>Male, n (%)	12 (66.7)	10 (62.5)	**0.064, 1 (0.800)**
**Hematological malignancy**, n (%)			
ALL	3 (16.7)	5 (31.2)	
AML/MDS	14 (77.8)	10 (62.5)	**3.060, 3 (0.383)**
CMML	1 (5.6)	0 (0)	
NHL	0 (0)	1 (6.2)	
**Disease status**, n (%)			
Not in remission	3 (16.7)	3 (18.8)	
Relapse	4 (22.2)	1 (6.2)	**1.732, 2 (0.421)**
Remission°	11 (61.1)	12 (75.0)	
**Type of HSCT**, n (%)			
Autologous	0 (0)	1 (6.2)	
Double cord blood	1 (5.6)	0 (0)	**2.267, 4 (0.687)**
Matched related	2 (11.1)	1 (6.2)	
Matched unrelated	3 (16.7)	3 (18.8)	
**Neutropenia***<br>n (%)	13 (72.2)	14 (87.5)	**1.209, 1 (0.271)**
**Graft-versus-host disease**<br>n (%)	5 (27.8)	4 (25.0)	**0.034, 1 (0.855)**
**Steroid use**<br>n (%)	5 (27.8)	3 (18.8)	**0.384, 1 (0.536)**
**CMV reactivation****<br>n (%)	3 (16.7)	4 (25.0)	**0.360, 1 (0.549)**

IMI, invasive mold infection; non-IFI, non-invasive fungal infection; ALL, acute lymphoid leukemia; AML, acute myeloid leukemia; MDS, myelodysplastic syndrome; NHL, non-Hodgkin's lymphoma; CMML, chronic myelomonocytic leukemia.

†This group includes invasive aspergillosis and mucormycosis patients. Remission defined as <5% blasts on bone marrow biopsy after induction chemotherapy. *Neutropenia defined as absolute neutrophil count (ANC) <0.5 x 10^9/L. **CMV reactivation defined as blood CMV DNA level in peripheral blood >100IU/ml in two occasions within the 12 months prior to data of sample collection.
