# Supplementary material for: Impaired T Cell Responsiveness to Interleukin-6 in Hematological Patients with Invasive Aspergillosis
Source: PLoS One. 2015 Apr 2;10(4):e0123171. doi: 10.1371/journal.pone.0123171 (PMC4383538; doi:10.1371/journal.pone.0123171)
Supplement: S3 Table — (PDF) [file pone.0123171.s009.pdf]

Supporting Table 3. White blood cell counts and mononuclear cell subpopulations for study patients							
	Peripheral blood cell counts (x 10^9/L)*				Number of gated events^		
**Study ID**	**Total WBC**	**Neutrophils**	**Lymphocytes**	**Monocytes**	**Monocyte**	**Memory CD4+ cells**	**Naïve CD4+ cells**
**IA_001**	7.5	5.6	0.53	0.53	280.00	1951	1514
**IA_002**	6.0	5.22	0.12	0.66	4133.00	1270	310
**IA_003**	1.6	1.12	0.02	0.37	2235.00	4621	380
**IA_004**	7.6	3.1	2.6	1.40	1028.00	1738	839
**IA_005**	7.2	4.63	1.98	0.55	2387.00	1760	339
**IA_006**	3.7	1.2	1.8	0.7	1016.00	1926	162
**IA_007**	4.8	2.4	1.7	0.6	1012.00	1192	395
**IA_008**	1.6	1.11	0.25	0.21	5144.00	1264	1992
**IA_009**	2.1	1.03	0.59	0.48	3443.00	2476	287
**IA_010**	1.2	1.13	0.02	0.05	2164.00	n/a	n/a
**IA_011**	7.2	n/a	n/a	n/a	n/a	19	121
**IA_012**	4.7	n/a	n/a	n/a	n/a	3026	176
**IA_013**	9.5	n/a	n/a	n/a	3889.00	3323	701
**IA_018**	10.2	8.57	0.1	0.31	2881.00	334	537
**Mean**	**5.35**	**3.19**	**0.88**	**0.53**	**2467.67**	**1915.38**	**596.38**
**SD**	**3.00**	**2.51**	**0.95**	**0.35**	**1486.79**	**1237.19**	**561.68**
**Non-IFI_001**	5.6	2.52	2.31	0.64	1597.00	737	611
**Non-IFI_002**	1.2	0.42	0.75	0.02	1708.00	3023	1601
**Non-IFI_003**	3.4	1.5	1.22	0.64	598.00	642	1113
**Non-IFI_004**	1.2	0.5	0.6	0	2312.00	840	150
**Non-IFI_005**	5.2	3.1	1.6	0.40	1055.00	2591	417
**Non-IFI_007**	0.5	0.03	0.43	0.01	n/a	1301	66
**Non-IFI_008**	4.1	2.63	1.1	0.28	1631.00	757	1690
**Non-IFI_011**	2.6	1.6	0.6	0.30	2921.00	1808	1330
**Non-IFI_015**	7.3	5.62	0.8	0.88	3092.00	2213	416
**Non-IFI_018**	10.1	7.02	1.74	1.27	1485.00	1498	2169

**Non-IFI_020**	1.0	0.2	0.5	0.20	1123.00	3498	2571
**Non-IFI_022**	4.5	3.6	0.7	0.20	1848.00	2407	3595
**Non-IFI_028**	4.1	3.3	0.5	0.20	1662.00	583	884
**Non-IFI_029**	9.5	9.32	0	0	1444.00	1050	1699
**Non-IFI_030**	7.5	4.2	1.58	1.43	4088.00	n/a	n/a
**Non-IFI_031**	6.2	3.8	1.3	1.10	2865.00	243	1732
**Mean**	**4.90**	**3.09**	**0.98**	**0.47**	**1961.93**	**1546.07**	**1336.27**
**SD**	**2.86**	**2.58**	**0.60**	**0.47**	**923.80**	**995.16**	**974.55**
**Mucor_001**	1.2	n/a	n/a	n/a	4433.00	1292	603
**Mucor_002**	6.7	5.23	0.69	0.67	2716.00	1293	2133
**Mucor_003**	2.1	0.28	0.72	0	319.00	n/a	n/a
**Mucor_004**	4	2.7	0.8	0.50	3071.00	2139	1093
**Mean**	**3.50**	**2.74**	**0.75**	**0.39**	**2634.75**	**1574.67**	**1276.33**
**SD**	**2.43**	**2.48**	**0.08**	**0.35**	**1712.05**	**488.73**	**781.30**
**T-Test (IMI vs Non-IFI)**	**0.97**	**0.99**	**0.67**	**0.85**	**0.23**	**0.43**	**0.051**
**T-Test (IA vs Non-IFI)**	**0.68**	**0.92**	**0.76**	**0.71**	**0.32**	**0.40**	**0.02**

IMI, invasive mold infection; IFI, invasive fungal infection; IA, invasive aspergillosis; SD, standard deviation; n/a not available. IMI group includes all IA and mucor cases. In the bottom, *p* values for comparison between early and late sample collection groups using unpaired T test.

*Peripheral white blood cell count measured by an automated cell counter at the time of sample collection is shown for each individual study patient. ^ Number of gated events in flow cytometry analyses for specific cell populations is shown for each individual patient. A total of 10,000 gated events based on forward Scatter (FSC) and side scatter (SSC) properties were collected for each sample. Dead cells were excluded based on FSC/SSC, and potential blast cells were excluded at the time of analysis by gating on CD45high cells. Monocytes, memory T helper and naïve T helper cells were defined as CD45highCD14+, CD45highCD3+CD4+CD45RO+ and CD45highCD3+CD4+CD45RO-, respectively.
