# Supplementary material for: Impaired T Cell Responsiveness to Interleukin-6 in Hematological Patients with Invasive Aspergillosis
Source: PLoS One. 2015 Apr 2;10(4):e0123171. doi: 10.1371/journal.pone.0123171 (PMC4383538; doi:10.1371/journal.pone.0123171)
Supplement: S4 Table — (PDF) [file pone.0123171.s010.pdf]

Supporting Table 4. Dectin-1 and pSTAT3 levels by time of sample collection *								
			Basal (pSTAT3, %)			Stimulated (pSTAT3, %)		
Study ID	Time from IMI to Sample Collection (Day)	Dectin-1 (%)	Monocytes	Memory T Helper Cells	Naïve T Helper Cells	Monocytes	Memory T Helper Cells	Naïve T Helper Cells
Early (<30 days from diagnosis of IA)								
**IA_001**	**11**	41.80	50.90	13.60	1.72	92.00	78.10	29.60
**IA_008**	**11**	26.70	0.32	14.70	14.70	70.60	83.40	82.30
**IA_009**	**2**	85.00	80.50	24.30	2.14	94.80	87.10	81.10
**IA_010**	**16**	51.80	-	-	-	-	-	-
**IA_012**	**11**	-	35.80	35.10	3.35	83.20	86.00	67.90
**IA_013**	**20**	60.20	48.00	39.00	62.20	92.80	93.20	97.80
**IA_018**	**23**	76.50	74.40	65.90	45.30	83.30	85.80	52.60
**Mean**	**13.43**	**57.00**	**48.32**	**32.10**	**21.57**	**86.12**	**85.60**	**68.55**
**SD**	**6.95**	**21.68**	**28.92**	**19.52**	**25.94**	**9.08**	**4.93**	**24.39**
Late (>30 days from diagnosis of IA)								
**IA_002**	**48**	34.3	86.4	56.2	37.7	79.4	69.5	79.6
**IA_003**	**42**	94.5	75.4	70.8	54.2	74.1	69.6	54.0
**IA_004**	**70**	91.7	3.32	1.21	1.55	80.5	63.2	22.4
**IA_005**	**57**	60.2	0.32	0.29	0.3	77.5	51.1	25.6
**IA_006**	**315**	86.3	6.17	0.99	3.09	69.9	52.0	35.7
**IA_007**	**389**	78.7	4.36	1.09	1.01	79.9	63.2	21.3
**IA_011**	**70**	-	50.4	5.26	1.65	79.7	73.3	52.7
**Mean**	**141.57**	**74.28**	**32.34**	**19.41**	**14.21**	**77.29**	**63.13**	**41.61**
**SD**	**145.70**	**23.12**	**37.50**	**30.46**	**22.21**	**3.92**	**8.70**	**21.57**
**T-test**	**0.0589**	**0.2114**	**0.4050**	**0.3853**	**0.5983**	**0.0649**	**0.0002**	**0.0624**

Supporting Table 4. Dectin-1 and pSTAT3 levels by time of sample collection (continued)						
	% Cells Fold Change (pSTAT3)			MFI Fold Change (pSTAT3)		
**Study ID**	**Monocytes**	**Memory T Helper Cells**	**Naïve T Helper Cells**	**Monocytes**	**Memory T Helper Cells**	**Naïve T Helper Cells**
Early (<30 days from diagnosis of IA)						
**IA_001**	1.81	5.74	17.21	2.90	4.04	3.01
**IA_008**	0.85	5.67	5.60	1.47	2.94	3.26
**IA_009**	1.18	3.58	38.62	3.36	4.52	3.88
**IA_010**	-	-	-	-	-	-
**IA_012**	2.32	2.45	20.27	3.74	3.88	2.54
**IA_013**	1.93	2.39	1.57	3.12	3.81	2.64
**IA_018**	1.12	1.30	1.16	1.58	1.68	1.62
**Mean**	**1.62**	**3.97**	**16.65**	**2.92**	**3.84**	**3.07**
**SD**	**0.59**	**1.66**	**14.54**	**0.87**	**0.57**	**0.54**
Late (>30 days from diagnosis of IA)						
**IA_002**	0.92	1.24	2.11	1.08	1.31	2.02
**IA_003**	0.98	0.98	1.00	1.07	1.08	1.03
**IA_004**	24.25	52.23	14.45	29.15	10.5	2.83
**IA_005**	-	-	86.78	-6.14	25.46	2.70
**IA_006**	1.33	52.68	11.55	-14.96	4.92	3.15
**IA_007**	18.33	57.98	21.09	-11.2	7.81	2.73
**IA_011**	1.58	13.94	31.94	3.29	1.94	3.19
**Mean**	**7.90**	**29.84**	**24.13**	**0.33**	**7.57**	**2.52**
**SD**	**10.54**	**27.27**	**29.63**	**14.43**	**8.65**	**0.76**
**T-test**	**0.200**	**0.064**	**0.447**	**0.680**	**0.259**	**0.489**

*For this time-dependent analysis, IA cases were divided in subgroups based on time of sample collection into early (study sample collected within 30 days of diagnosis) and late (study sample collected after 30 days of diagnosis). Levels of Dectin-1 expression on monocytes, and basal and IL-6 induced pSTAT3 levels on monocytes, memory and naïve T helper cells are shown. Fold change was calculated by dividing the percentage or mean fluorescence intensity (MFI) of pSTAT3 in stimulated cells by that of unstimulated cells. In the bottom, *p* values for comparison between early and late sample collection groups using unpaired T test.
